# Obstructive Colloid Cyst of the Third Ventricle

**DOI:** 10.5334/jbr-btr.1306

**Published:** 2017-04-25

**Authors:** Charlemagne Noukoua

**Affiliations:** 1 André Renard hospital liège, BE

**Keywords:** Hydrocephalus, colloid cyst, foramen of Monro

A 44-year-old female was admitted to the emergency department complaining of gradually worsening headache over the previous few days, and a recent episode of amnesia. On admission, the clinical and neurological examinations were normal. The patient underwent unenhanced head computed tomography (CT) that revealed hydrocephalus and indirect signs of intracranial hypertension, with hypodensities around the cerebral ventricles (arrow) indicating transependymal migration of the cerebrospinal fluid (CSF) (Figure 1). A round structure, isodense relative to brain (open arrow), with a tiny calcification (arrowhead) was found in the third ventricle. As this led to the suspicion of obstructive colloid cyst, brain magnetic resonace imaging (MRI) was carried out (Figure 2). It confirmed the previous CT findings, consisting of acute hydrocephalus with fluid migration (hyperintensities on fluid attenuated inversion recovery (FLAIR) T2-weighted images) around the ventricles and a nodular lesion (with fluid signal on T2-weighted images and intermediate signal on T1-weighted images) on the roof of the third ventricle, obstructing the foramen of Monro. The patient was admitted to the neurosurgical department for surgical excision.

**Figure 1:**
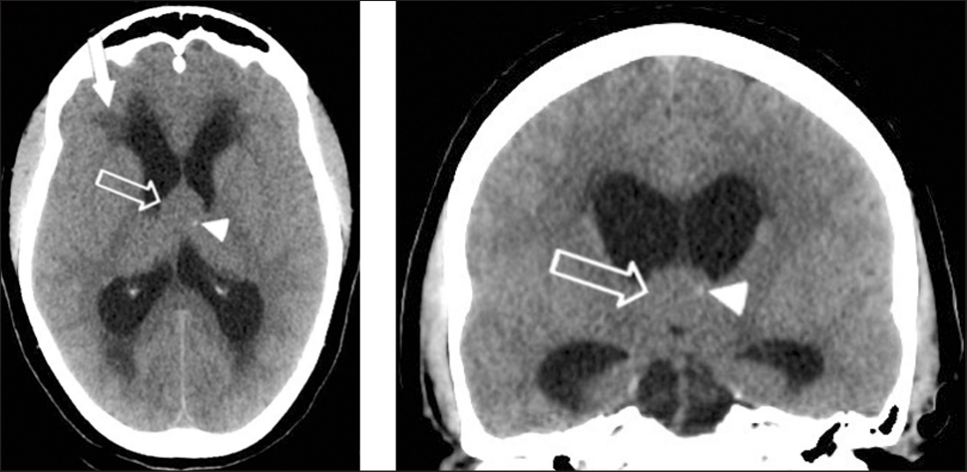


**Figure 2:**
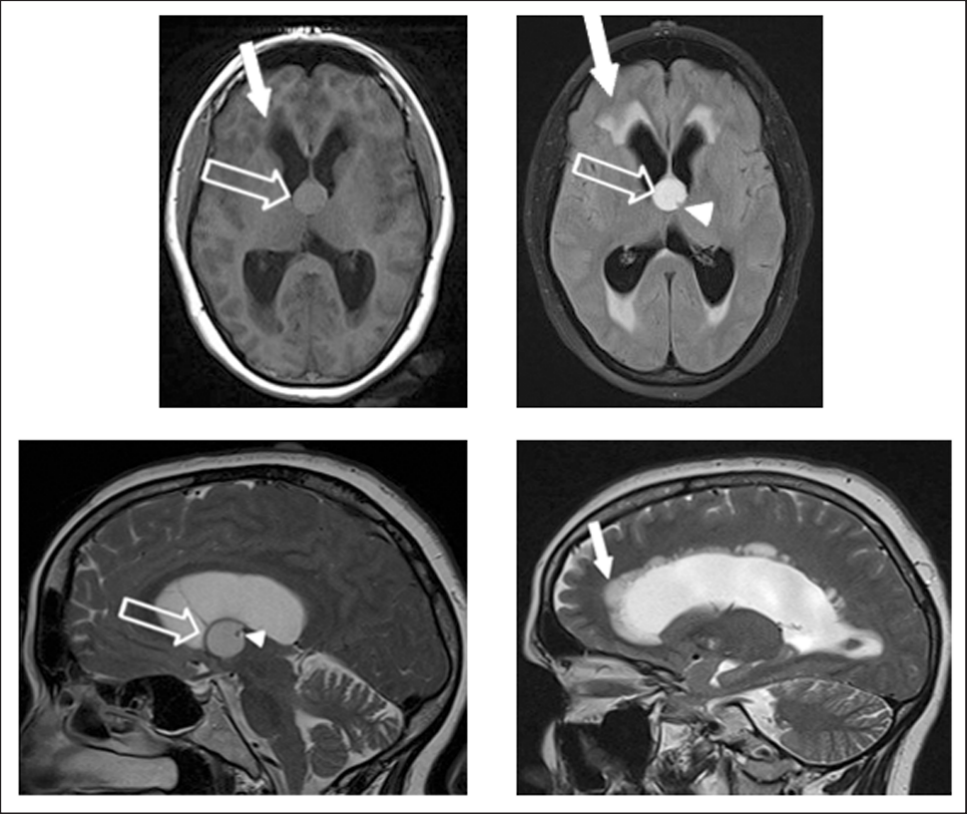


## Comment

Colloid cysts of the third ventricle are benign tumors that accounts for 0.5–3% of primary brain tumors. As such, they can only be seen using cross-sectional imaging techniques. Most cases are found incidentally in middle age (the fourth decade), though some may be rapidly symptomatic and diagnosed in the pediatric age. The etiology of colloid cysts is still unclear. Their inner walls are lined with a single layer of epithelium that produces mucin, this ultimately determines the imaging properties of the cyst. On CT, the variable density of cysts depends on the respective proportion of fluid, cholesterol and protein within the cyst. Occasionally, the cyst misleadingly appears hypodense or isodense compared to the brain as in this case. Similarly, the signal of the cyst on MRI is variable [1]. They are usually hyperintense on T1-weighted sequences and hypointense on T2-weighted sequences. In these cases, they may be difficult to distinguish from the surrounding CSF on FLAIR sequences. When the amount of cholesterol and protein is low, the signal intensity on T1- and T2-weighted sequences is similar to the CSF. In these conditions, FLAIR sequences are most helpful. These lesions may therefore go undiagnosed in neonates to whom FLAIR sequences are not routinely applied. Diffusion-weighted and contrast-enhanced sequences are often unremarkable. Of note, calcification in the wall of the cyst, as in the presented case, is uncommon.

The cysts are usually located on the roof of the anterior side of the third ventricle, between the fornices and behind the foramen of Monro. They may undergo slow growth due to the production of mucin. The subsequent displacement of these cysts towards the foramen of Monro causes obstruction to the CSF circulation, leading to acute hydrocephalus and raised intracranial pressure. The clinical presentation is non-specific but includes intermittent headaches, nausea, vomiting, loss of consciousness, amnesia and drop attack. Treatment of colloid cyst includes stereotaxic aspiration, endoscopic aspiration and microsurgical procedures. To this regard, the following characteristics may provide important information to the surgeon for the procedure of choice: hypodensity on CT and hyperintensity on T2-weighted sequences indicate low viscosity and suitability of stereotaxic aspiration.
